# Combined Effects of Fludarabine and Interferon Alpha on Autophagy Regulation Define the Phase of Cell Survival and Promotes Responses in LLC-MK2 and K562 Cells

**DOI:** 10.3390/medsci10010020

**Published:** 2022-03-17

**Authors:** Pathompong Bowornruangrit, Supeecha Kumkate, Wipawan Sirigulpanit, Vijittra Leardkamolkarn

**Affiliations:** 1 Department of Anatomy, Faculty of Science, Mahidol University, Bangkok 10400, Thailand; astroboss@gmail.com; 2 Department of Biology, Faculty of Science, Mahidol University, Bangkok 10400, Thailand; supeecha.kum@mahidol.ac.th; 3 Division of Pharmacology and Biopharmaceutical Sciences, Faculty of Pharmaceutical Sciences, Burapha University, Chonburi 20131, Thailand; wipawans@go.buu.ac.th

**Keywords:** autophagy, fludarabine, interferon-alpha, K562, LLC-MK2, STAT1

## Abstract

Autophagy is a known mechanism of cells under internal stress that regulates cellular function via internal protein recycling and the cleaning up of debris, leading to healthy live cells. However, the stimulation of autophagy by external factors such as chemical compounds or viral infection mostly tends to induce apoptosis/cell death. This study hypothesizes that manipulation of the autophagy mechanism to the pro-cell survival and/or decreased pro-viral niche can be a strategy for effective antiviral and anticancer treatment. Cells susceptible to viral infection, namely LLC-MK2, normal monkey epithelium, and K562, human immune-related lymphocyte, which is also a cancer cell line, were treated with fludarabine nucleoside analog (Fdb), interferon alpha (IFN-α), and a combination of Fdb and IFN-α, and then were evaluated for signs of adaptive autophagy and STAT1 antiviral signaling by Western blotting and immunolabeling assays. The results showed that the low concentration of Fdb was able to activate an autophagy response in both cell types, as demonstrated by the intense immunostaining of LC3B foci in the autophagosomes of living cells. Treatment with IFN-α (10 U/mL) showed no alteration in the initiator of mTOR autophagy but dramatically increased the intracellular STAT1 signaling molecules in both cell types. Although in the combined Fdb and IFN-α treatment, both LLC-MK2 and K562 cells showed only slight changes in the autophagy-responsive proteins p-mTOR and LC3B, an adaptive autophagy event was clearly shown in the autophagosome of the LLC-MK2 cell, suggesting the survival phase of the normal cell. The combined effect of Fdb and IFN-α treatment on the antiviral response was identified by the level of activation of the STAT1 antiviral marker. Significantly, the adaptive autophagy mediated by Fdb was able to suppress the IFN-α-mediated pSTAT1 signaling in both cell types to a level that is appropriate for cellular function. It is concluded that the administration of an appropriate dose of Fdb and IFN-α in combination is beneficial for the treatment of some types of cancer and viral infection.

## 1. Introduction

Autophagy is a cellular system that protects cells from significant stress stimuli such as starvation and infection. The mechanism of autophagy involves the segregation of cytoplasmic proteins and organelles, as well as the lysosomal degradation pathway. The stress stimulation induces a protein called mammalian target of rapamycin (mTOR) to mediate phosphorylation of the downstream enzymatic system, whose activity is sustained during a normal state to sequential activation of several proteins including Beclin-1, Atg14L, and Atg8 (or microtubule light chain 3B (LC3B)). The activated LC3B (LC3B-I) is converted to LC3B-II then conjugated with phosphatidylethanolamine, and subsequentlypinned onto a sprouted membrane of the endoplasmic reticulum. This membrane structure continues to expand to form an autophagosome and is translocated along the microtubule network to fuse with the lysosome, then turns into an autolysosome. The autophagosome plays roles in self-digestion and the recycling of useful proteins for cell metabolism, as well as in self-clean-up of the damaged or injured materials within the cells [1]. Besides playing a role in the homeostatic mechanism of the cells, autophagy acts as an effector arm of the immune system by sequestering intracellular pathogens to prime CD8^+^ T cells, increasing phagocytosis (innate immunity) and stimulating cytokine production that regulates T and B lymphocytes’ maturation and survival (adaptive immunity). Accumulated data have shown that autophagy involves a large amount of cell signaling and the production of many cytokines. For example, interleukin-1 (IL-1), IL-2, IL-6, tumor necrosis factor (TNF-α), transforming growth factor (TGF-β), and interferon (IFN-γ) have been shown to be effectors of autophagy induction, whereas IL-4, IL-10, and IL-13 have been shown to be effectors of autophagy inhibition [2]. The over-production of these pro-inflammatory cytokines, called a “cytokine storm”, seems to play a central role in the progression and exacerbation of disease by leading to the recruitment of immune cells to infection sites. However, the signaling interaction of IFN-α with autophagy modulation and response in lymphocytes has not been explored. It might be a complicated interaction and possibly involves crosstalk between each party in order to regulate the immune system [3]. Autophagy is also involved in tumorigenesis via stimulated autophagic cell death. However, in some conditions, autophagy also promotes tumor cell survival [4]. This important aspect requires further investigation for advancing the field of therapeutic strategy.

Fludarabine (Fdb) or fludarabine monophosphate (an analog of purine nucleoside, fluorinated arabinosyl-adenine-AMP, and F-ara-AMP) is one of the drugs developed for the treatment of cancers [5,6,7,8,9,10]. Fdb’s known mechanism of action is through the disturbance of DNA replication and cell cycle arrest. Fdb also inhibits adenosine deaminase, leading to a lack of metabolites for cell survival and the emergence of the process of apoptosis/cell death [11]. Currently, Fdb is specified for the treatment of B-cell chronic lymphocytic leukemia in patients who have not responded to traditional therapy. It has been used for treatment of non-Hodgkin’s lymphoma, hairy cell leukemia, cutaneous T-cell lymphoma, and acute myeloid leukemia. The major toxicity at the current clinical doses is transient myelosuppression and immunosuppression with an increased risk of opportunistic infections. The common side effects for patients taking Fdb are temporarily decreased blood counts, anemia, and/or bleeding, with some individuals having pulmonary system toxicity related to fluid. Since a single Fdb treatment has a highly cytotoxic effect, the combination of Fdb with other anticancer drugs is widely used, especially for lymphoma cancer therapy [12]. However, a combined treatment with Fdb and rapamycin to primary chronic lymphocytic leukemia cells has shown a higher toxicity, which correlates with Bcl-2 expression [13]. Therefore, balancing the therapeutic effects with toxicity control is considered during the development of the new acceptable medicinal recipe [14]. Lately, drug development research has raised Fdb as an effective antiviral drug since Fdb has shown an inhibitory effect against emerging RNA virus infections, including positive-stranded RNA viruses, Zika virus, enterovirus, and negative-strand RNA severe fever with thrombocytopenia syndrome virus that was not cell type-dependent [15]. Moreover, research has shown that Fdb can inhibit the expression of the severe acute respiratory syndrome (SARS-CoV-2, coronavirus-2019, COVID-19) receptor via STAT1 signaling in respiratory epithelial cell lines [16]. In such research, Fdb not only had a direct antiviral effect, inhibiting viral RNA replication and protein expression, but its chemical properties, such as anti-nucleotide replication, anti-proliferation, and apoptosis induction, interfered with cell survival as well as the viral life cycle.

Interferons (IFNs), including IFN-α, IFN-β, IFN-ε, IFN-κ, and IFN-ω, are cytokines that display multiple properties, especially tumor suppression and antiviral activities. In terms of anticancer activity, the exact mechanism of action of IFNs is unclear, but they can arrest cell growth [17]. No specific genes have been linked to the anti-proliferative activity of IFNs; however, STAT1 is believed to be involved since it is often deficient in tumors [18]. Clinically, the recombinant IFN-α has been used effectively in the treatment of certain hematologic malignancies, including hairy cell leukemia, chronic myeloid leukemia, multiple myeloma, and AIDS-associated Kaposi sarcoma [19]. It is also used to enhance the effectiveness of chemotherapy in some cancers. IFN-α administration for cancer treatment is of benefit in terms of both its direct anti-angiogenic property via the downregulation of basic fibroblast growth factor (bFGF) expression and the indirect inhibition of tumor cells via the immune system [20]. However, IFN-α treatment is considered toxic since it may lead to exacerbations of autoimmune disorders. IFN-α side effects, including excessive suppression of blood counts, retinopathy, sarcoidosis, and left-sided heart failure, are time- and dose-dependent [21]. Whether or not and how IFN-α is involved in autophagy induction is under investigation in the current research. A study of the K562 cell line revealed that IFN-α could stimulate autophagic flux via STAT1 signaling [22]. In terms of antiviral activity, immune regulation by interferon treatment has been recognized and clinically applied. A prominent example of treatment involves IFN-α, which has been used successfully as a first-line and/or co-treatment with other drugs such as ribavirin for patients susceptible to chronic viral hepatitis B and hepatitis C infection [23]. Moreover, administration of IFN-α is considered worthwhile against other human life-threatening viruses, including human immunodeficiency virus (HIV) and emerging viruses such as some coronaviruses. Although studies of patients infected with HIV indicate that IFN-α could not inhibit HIV latency and may cause neurotoxicity [24,25], IFN-α treatment demonstrated a profound event of immunity stimulation, especially in cell-mediated immune responses of HIV-infected cells [26,27]. During the recent COVID-19 pandemic, IFN-α has received considerable attention as an anti-COVID-19 treatment. Administration of IFN-α to COVID-19-infected patients has demonstrated that IFN-α could reduce COVID-19 disease severity [28]. However, given the limitations of the insights of the scientific investigations and the unclear treatment period that correlated with side effects and therapeutic efficiency, the data are still insufficient in terms of defining whether IFN-α could be a choice to potentiate strategies for anti-COVID-19 treatment. In addition, whether IFN-α engages in antiviral activities during early infection in the acute stage is still unknown.

At present, research strategies aiming for the treatment of incurable diseases such as cancers and viral infections are focusing on the combined treatment of existing drugs to increase effectiveness and reduce the undesired side effects of the drugs. These could be performed in cell-cultured models using susceptible cell lines to investigate the roles and mechanistic interaction of drugs such as Fdb and IFN-α, which are used in this study. In this study, we modified the general cell culture technique with immunohistochemistry and a confocal microscopy study to obtain more meaningful and supportive results for the evaluation of the intracellular changes in live cells such as autophagy, as well as noting other specific phenomena that occurred during the drug treatment. The specific aim of this study was to prove that autophagy stimulation with an appropriate dose of Fdb (which is pro-cell survival, not pro-cell death) benefits the understanding of the antiviral and/or anticancer activities of IFN-α treatment.

## 2. Materials and Methods

### 2.1. Cell Culture

LLC-MK2, the rhesus monkey kidney epithelial cell line, and the K562 human leukemic cell line were purchased from ATCC (Manassas, VA, USA), propagated, and stocked at −80 °C. LLC-MK2 cells were cultured in Dulbecco’s Modified Eagle Medium (DMEM) supplemented with 10% fetal bovine serum (FBS) and penicillin–streptomycin antibiotic mixture (HyClone^®^, Logan, Utah, USA) at 37 °C in a 5% CO_2_ incubator. K562 cells were cultured in RPMI-1640 media (HyClone^®^, Utah, USA) supplemented with 10% FBS and the antibiotic mixture. Trypsin–EDTA solution was used for the detachment of LLC-MK2 cells.

### 2.2. Chemicals and Antibodies

Fludarabine phosphate (3495, Abcam^®^, Cambridge, UK) was dissolved in dimethyl sulfoxide (DMSO) (D8418, Sigma-Aldrich^®^, Darmstadt, Germany) at 100 mM for stock solution and stored at −20 °C. The stock was diluted in the culture media for specific treatment. Human recombinant interferon alpha A/D (IFN-α) (I4401, Sigma-Aldrich^®^, Darmstadt, Germany) was diluted to 100 U/mL in the culture media directly before the treatment. Methylthiazolyldiphenyl-tetrazolium bromide (MTT) (M6494, ThermoFisher^®^, Waltham, MA, USA) powder was dissolved in phosphate-buffered saline (PBS) and added to the prepared stock solution (12 mM). The solution was filtered through a 0.2 µm membrane and stored at −20 °C before it was used in the MTT assay. Polyclonal antibodies against mTOR (2972S), p-mTOR (2971S), Beclin-1 (3738S), LC3B-I and II (2775S), monoclonal antibodies against p-STAT1 (9167S), and β-actin (4970S), HRP-conjugated (7074S) and Alexa^®^ 555 conjugated (4413S) antibodies were commercially supplied from Cell Signaling^®^ (Danvers, MA, USA).

### 2.3. MTT Assay to Determine the Cytotoxic Effect of Fdb

LLC-MK2 and K562 cells were grown to 95% confluence in a T75 culture flask (Corning^®^, Corning, NY, USA). The cells were plated onto a 96-well plate (Corning^®^, New York, NY, USA) (100 µL per well for 10,000 cells) and cultured for 48 h. The cells were treated with a 10-fold diluted concentration series (100.0, 10.0, 1.0, 0.1, and 0.01 µM) of Fdb in culture media plus 0.0001% DMSO. The non-treatment control group was treated with culture media plus 0.0001% DMSO. The treated cells were incubated at 3 °C in a 5% CO_2_ incubator for 24 and 72 h (for LLC-MK2 cells) or 96 h (for K562 cells). MTT solution was added (1:10 by volume) and incubated until the purple formazan crystal formed. The crystal product was solubilized by MTT solubilizing agent (80% isopropanol and 10% Triton X-100 in 1.0 N HCl). The product was measured in terms of optical absorbance at 562 nm by a microplate spectrometer. The data were quantified and a inhibition concentration index of 50% cell viability (IC_50_) was determined by non-linear regression analysis using GraphPad^®^ Prism 5 software.

### 2.4. Biochemical and Immunohistochemical Analysis for Autophagy and Immune-Signaling Response

K562 and LLC-MK2 cells were seeded onto 6-well plates (1 mL for 10,000 cells) and grown to 95% confluence. The cells were treated with the desired concentrations of the following agents: (1) Fdb freshly prepared from stock in culture media with 0.001% DMSO, (2) IFN-α prepared in culture media, (3) combined IFN-α and Fdb in culture media with 0.001% DMSO, (4) non-treatment control (culture media with 0.001% DMSO). All treatments were performed in triplicate.

#### 2.4.1. Western Blot Analysis

After treatment, the cells were collected in suspension. The pellets were washed with PBS and lysed, then extracted in a RIPA lysis buffer containing protease and phosphatase inhibitors (ThermoFisher^®^, Waltham, MA, USA). Total protein was collected and quantified by a BCA assay kit (Merck^®^ 71285), and we measured the absorbance at 562 nm on a plate reader. A total of 30 µg of protein was denatured with SDS solution and loaded into 8–15% acrylamide gel. The protein was electrophoresed at 75 V for 150 min in a Tris-glycine-SDS buffer. Then, they were transferred to nitrocellulose membrane at 100 V for 90 min. The transferred membrane proteins were processed to immunostaining at 4 °C overnight with shaking. The membranes were incubated with 3% bovine serum albumin (BSA) solution in Tris buffer with 0.1% Tween-20 (TBST) as a blocking solution. Then, they were replaced with the primary antibody of each target protein, prepared in blocking solution (1:1000 dilutions of the primary antibodies against p-mTOR, mTOR, Beclin-1, LC3B-II, pSTAT1, and β-actin), and incubated overnight. The membranes were washed with TBST buffer and the anti-primary antibodies were replaced with 1:2000 dilution of secondary antibody-conjugated HRP. The ECL HRP substrate solution (1721064, BIO-RAD^®^, Hercules, CA, USA) was added and developed on Hyperfilm™ (GE Healthcare^®^, Chicago, IL, USA). Each film picture was digitized to TIFF file format and the relative density of the protein reaction was semi-quantified by the ImageJ^®^ program. Each experiment was performed in triplicate. The statistical analysis was run by GraphPad^®^ Prism 5 software.

#### 2.4.2. Immunohistochemical Analysis

The live-cell immunofluorescence staining method was applied to demonstrate autophagosome formation in the treated cells. The treatment process was the same as in the Western blot analysis discussed above, but the cells were plated onto a Chamber Slide™ kit (C7182, Nunc^®^, Roskilde, Denmark). After 24 h of treatment, the treated cells were fixed in absolute methanol for 15 min at −20 °C, then permeabilized with 0.1% Triton X-100 in PBS for 20 min, washed, and incubated in 3% BSA with PBS as a blocking solution for 2 h at room temperature. The cells were incubated with primary antibody against LC3B (1:1000 dilution in blocking solution) overnight at 4 °C. The secondary antibody-conjugated Alexa^®^ 555 (1:2000 dilution) was added and the mixture was incubated in the dark for 1 h at room temperature. The slides were washed by blocking solution with nuclear stain DAPI, mounted by glycerol, and sealed by nail polish. The fluorescent illumination was observed under a confocal microscope (Olympus^®^ FV10i-DOC).

## 3. Results

### 3.1. Cell Viability Induced by Fdb

**Figure 1:**
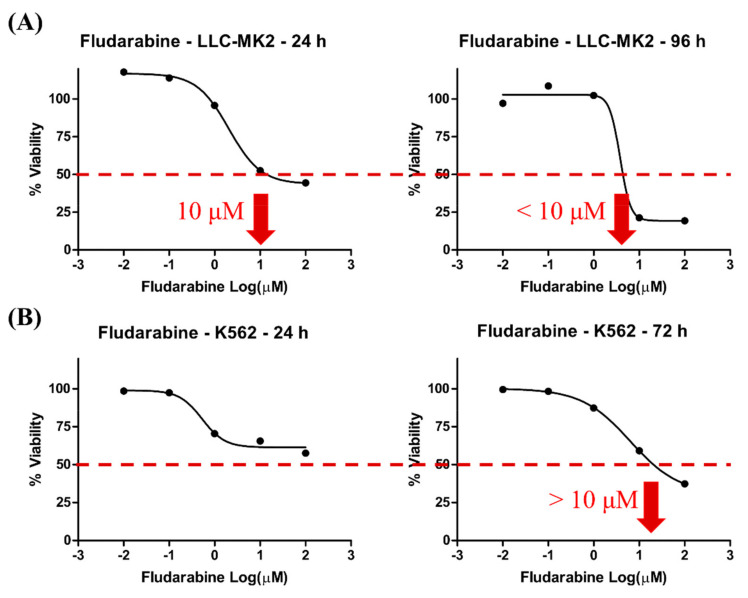
The time- and concentration-dependent effects of Fdb on (**A**) LLC-MK2 and (**B**) K562 cells. The cultured cells were treated with Fdb concentrations of 0, 2.5, 5, and 10 µM. Percentage of cell viability was determined after 24, 72, and 96 h of incubation at 37 °C in a 5% CO_2_ incubator by MTT assay. The dotted line indicates axis intercepts of IC_50_ and the log (dose) estimating value.

The MTT assay showed a low-dose, non-toxic concentration of Fdb (less than 10 µM) in both LLC-MK2 and K562 cells. The inhibitory concentration that reduced 50% cell viability (IC_50_) of Fdb in LLC-MK2 cells was approximately 10 µM and in K562 cells was >10 µM (Figure 1). The results suggested that the LLC-MK2 cell is more sensitive to Fdb treatment than the K562 cell. Therefore, the selected highest concentration used in this study was <10 µM to avoid apoptosis.

**Figure 2:**
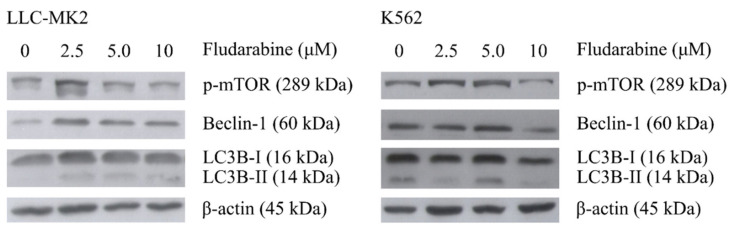
Western blot analysis of protein expression in LLC-MK2 and K562 cell treatment with Fdb (concentrations 0, 2.5, 5, 10 µM) for 24 h. The total cellular proteins were extracted and transferred to nitrocellulose membranes. The membranes were immunostained with specific antibodies against the autophagy-related proteins p-mTOR, Beclin-1, and LC3B-I/LC3B-II and processed for reaction color development. β-Actin was used as an internal control.

**Figure 3:**
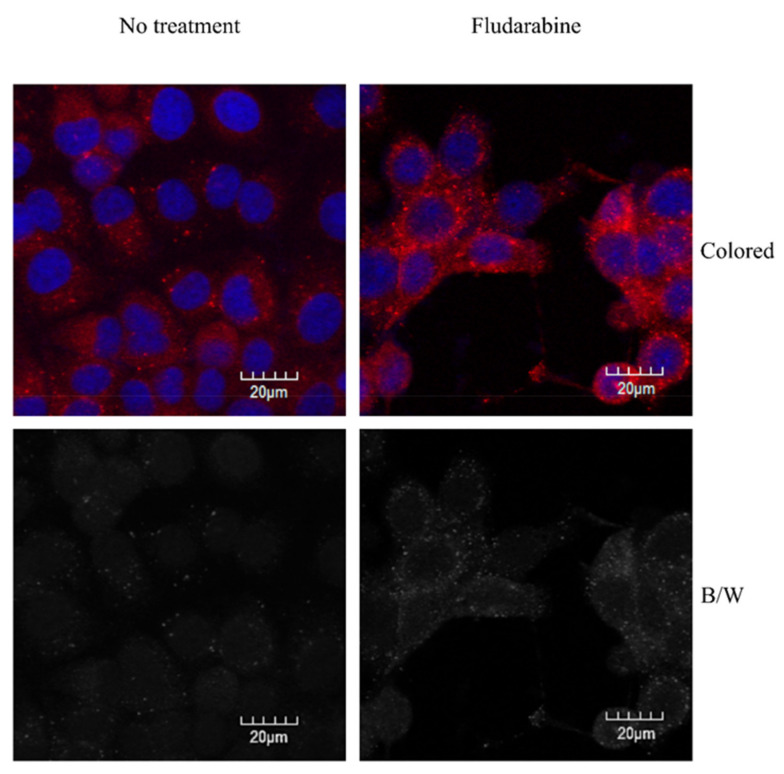
Confocal immunofluorescent photomicrographs taken from confocal microscopy of LLC-MK2 cells after 24 h of treatment with Fdb (5.0 µM). The treated cells were briefly fixed in absolute methanol at −20 °C, permeabilized with 0.1% Triton X-100, and then immunohistochemically stained with autophagy-activated protein (LC3B-I/LC3B-II) antibody and secondary antibody-conjugated Alexa^®^ 555. The fluorescent illumination of LC3B-II foci (autophagosome) was demonstrated in the cytoplasm of the cells. LC3B Alexa^®^-stained = red, DAPI nuclear-stained = blue.

Western blot analysis revealed that Fdb treatment (24 h) did not significantly alter the autophagy initiator, mTOR, and its subsequent activator, LC3B, in both LLC-MK2 and K562 cells, except that it slightly increased Beclin-1 (Atg6) (anti-apoptosis) in LLC-MK2 cells and slightly decreased Beclin-1 in K562 cells (Figure 2). However, the autophagy-activated protein (LC3B-II) was demonstrated in LLC-MK2 live cells by intense immunostaining of LC3B-II foci (autophagosome) in the cytoplasm (Figure 3). Attempts to perform K562 live-cell immunostaining were not successful due to limitations of adhering cells to the slides, most likely leading to death of the cells.

### 3.2. IFN-α Induced STAT1 Signaling in Both LLC-MK2 and K562 Cells That Rendered IFNR

**Figure 4:**
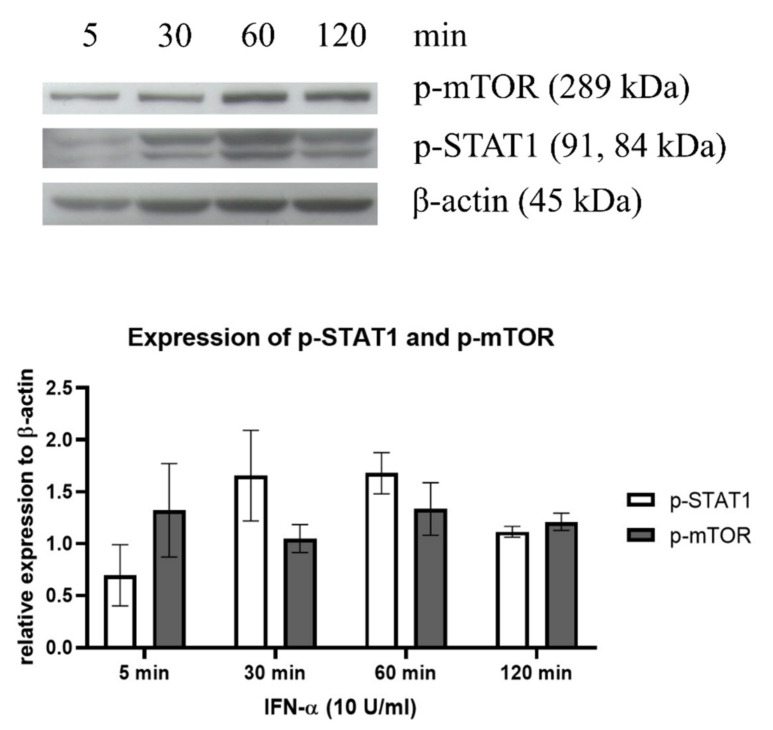
Western blot analysis of an autophagy inducer (p-mTOR) and the signaling activator (p-STAT1) protein expression in representative cells that rendered IFNR after treatment with IFN-α. LLC-MK2 cells were treated with IFN-α (10 U/mL) for 5, 30, 60, and 120 min. The cellular total proteins were extracted and transferred to nitrocellulose membranes. The membranes were immunostained with p-mTOR and p-STAT1 antibodies and processed for reaction color development. β-Actin was used as an internal control. The experiment was performed in triplicate and the band density was normalized with β-actin. Protein electrophoresis films were digitized and the relative density level of p-mTOR and p-STAT1 were measured by ImageJ^®^ software.

Western blot analysis demonstrated that IFN-α (10 U/mL) did not significantly induce an autophagy response through mTOR but slightly increased p-STAT1 signaling molecules in the treated LLC-MK2 cells, suggesting that there was no direct relationship between mTOR-mediated autophagy activation and the IFN-α-mediated p-STAT1 signaling pathway (Figure 4).

### 3.3. The Effect of Fdb Treatment on IFN-α-Exposed LLC-MK2 and K562 Cells

Cultured LLC-MK2 and K562 cells were exposed for 60 min to IFN-α (10 U/mL) before treatment with Fdb (10 μM) for 24 h. The cells were evaluated for autophagy response and antiviral response.

**Figure 5:**
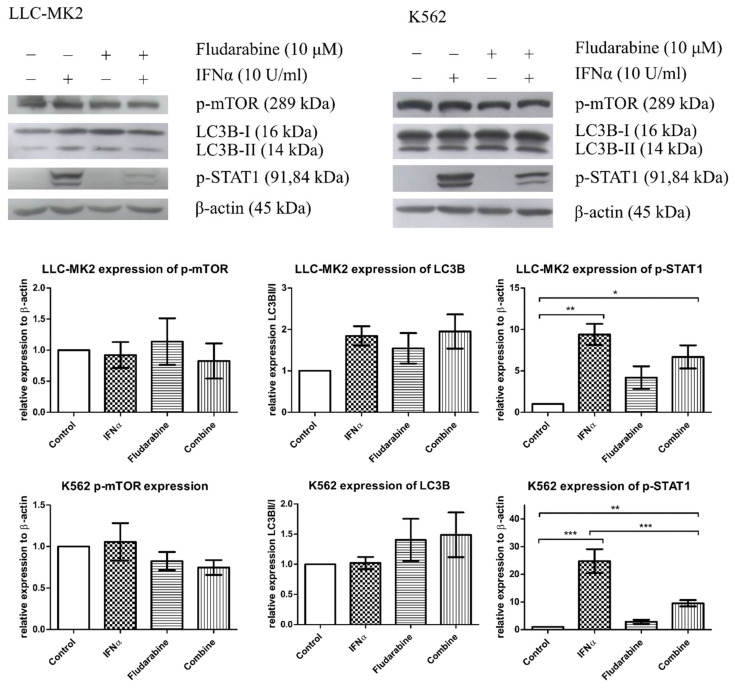
Western blot analysis of autophagy response and the antiviral response in cultured LLC-MK2 and K562 cells that were exposed for 60 min to IFN-α (10 U/mL) before treatment with Fdb (10 μM) for 24 h. The cells were evaluated for autophagy response mediation via p-mTOR and LC3B-I/LC3B-II activation, as well as antiviral response via p-STAT1 signaling molecules. The total proteins were extracted and transferred to nitrocellulose membranes. The membranes were immunostained with p-mTOR, LC3B-I/LC3B-II, and p-STAT1 antibodies and processed for reaction color development. β-Actin was used as an internal control. Protein electrophoresis films were digitized and the relative density level of p-mTOR (normalized with β-Actin), autophagosome marker LC3B-II (normalized with LC3B-I), and p-STAT1 (normalized with β-Actin) were measured by using ImageJ^®^ software. One-way ANOVA statistical analysis was performed (*, **, *** indicates a *p*-value of <0.05, 0.01, 0.001, respectively). The experiments were analyzed in triplicate and SEM is marked by an error bar.

Western blot analysis showed slightly altered levels of autophagy-responsive proteins and activation of p-mTOR and LC3B-I/LC3B-II in both LLC-MK2 and K562 cells receiving IFN-α or Fdb treatment. However, IFN-α exposure significantly increased the p-STAT1 antiviral response in both cell types. Fdb treatment in IFN-α-exposed LLC-MK2 and K562 cells suppressed the p-STAT1 activation level from IFN-α enhancement, but the level was still significantly higher in comparison with that of the untreated cells (Figure 5). It is worth noting that the inhibitory effect of Fdb to IFN-α-mediated p-STAT1 signaling was stronger in K562 cells than in LLC-MK2 cells.

### 3.4. Autophagy Phenomena in Live Cells after Treatment with IFN-α, Fdb, and IFN-α + Fdb

**Figure 6:**
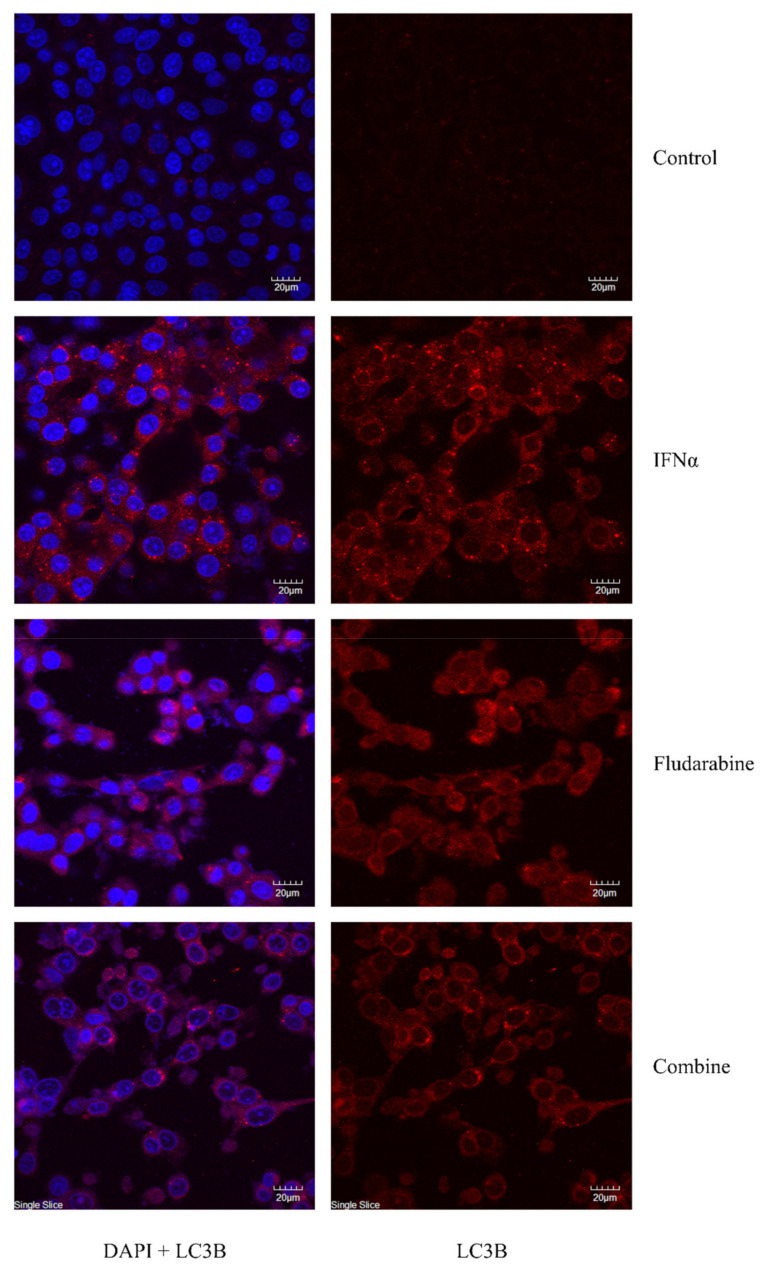
Confocal immunofluorescent micrographs of LLC-MK2 cells after exposure to IFN-α (10 U/mL) for 60 min before treatment with Fdb (10 μM) for an additional 24 h. The cells were briefly fixed in absolute methanol at −20 °C and permeabilized with 0.1% Triton X-100 before being immunohistochemically stained with autophagy-activated protein (LC3B-I/LC3B-II) antibody and secondary antibody-conjugated Alexa^®^ 555. The fluorescent illumination demonstrated the clustering of LC3B foci or autophagosome formation in the cytoplasm of the cells. LC3B Alexa^®^-stained = red, DAPI nuclear-stained = blue.

Immunohistochemical analysis of LLC-MK2 live cells pre-incubated with IFN-α (10 U/mL) for 60 min before receiving Fdb (10 μM) treatment for an additional 24 h demonstrated increased LC3B-II immunostaining foci (autophagosome) in the cytoplasm of the cells in comparison with the untreated control. The amount of cell loss was approximately equal in cells exposed to IFN-α alone, cells that received Fdb alone, and cells that received Fdb treatment after IFN-α exposure, as compared to the untreated control (Figure 6). Note that the positive immunostained cells were similar in terms of morphological changes, from spindle shapes to round shapes with condensed nuclei, suggesting the ongoing process of autophagosome formation, as well as functioning in order to prevent the effect of cell death, also known as adaptive autophagy.

## 4. Discussion

According to the immunolabeling results, Fdb at the concentration used in this experiment (10 µM or less) could induce an autophagy response in LLC-MK2 and K562 cells through the activation of the autophagy-controlling protein mTOR and its downstream LC3B-I/LC3B-II conversion, leading to adaptive cell survival. The immunofluorescent images of Fdb-treated cells clearly presented autophagy response in the form of autophagosome aggregation. Western blotting analysis revealed a slightly increased level of Beclin-1 (Atg6), an anti-apoptotic protein, in the epithelium of Fdb-treated cells, whereas Beclin-1 was unaltered in lymphocytes, which confirmed the strength of blood cells that were resistant to caspase-dependent programmed cell death, as has been shown in anti-leukemic conditions [4,7,9]. The induced active autophagy responses in LLC-MK2 cells reflected the reduced the metabolism and self-cleaning of materials inside the cell, leading to compromised cell survival [29,30]. However, in the cancer or immune-related cell K562, Fdb-induced autophagy targeted the response on an alternative pathway to the signal regulation that provides the cell’s pre-conditions for antiviral activity [31]. Previously, autophagy mediated by Fdb has been mentioned as a mechanism for drug resistance during chemotherapy, which implies sustained viability of the cells [32,33,34]. In this study, autophagy response in both cell types displayed the survival stage since we witnessed an adaptive event of the cells that underwent stress conditions from the drug. Fdb has only shown cytotoxicity to these cells at high doses, indicating that it could be safe for future clinical applications.

IFN-α significantly induced p-STAT1 signaling in both the epithelial cell (LLC-MK2) and the lymphocyte (K562) since both cells rendered the interferon receptor (IFNR). The p-STAT1 signaling level was approximately two- to three-fold greater in K562 cells than in LLC-MK2 cells. This was dependent on the nature of the cells, as stimulation of IFNR mediates signal transmission through the JAK/STAT pathway and/or the other cellular pathways that control cell behavior [35]. The induced STAT1 signaling mediator for antiviral activity of the immune-related K562 cell directly targeted the effectors of the JAK/STAT pathway that induces interferon-stimulated genes (ISGs) and stimulates subsequent antiviral gene expression for innate immunity response [36,37]. These gene products inhibit viral replication by interfering with the transcription of viral nucleic acid and impeding viral dissemination. In the non-immune cells such as LLC-MK2, STAT1 signaling could be cross-talked in order to activate autophagy-controlling molecules that are processed for further mechanisms that contribute to survival and growth of the cells or to inhibition of the process towards apoptosis/cell death [38].

The assessment of the benefits of Fdb to potentiate an antiviral effect of IFN-α in the immune-related lymphocyte K562 revealed that Fdb could suppress IFN-α-induced STAT1 protein expression and activity to the level that was shown by the untreated control suggesting that the combined effects between the two agents could reduce the undesired effect of IFN-α. The results clearly demonstrated that the appropriate dose of Fdb could alleviate IFN-α-induced STAT1 signals for antiviral response and that the STAT1 signaling in this cell type has a cross-talk activity with autophagy proteins, resulting in adaptive cell survival. Previously, research has raised the point that in the cells that render virus infection, the autophagy response can promote viral replication and propagation, leading to lytic cycle or induced cell death [39,40,41]. Thus, for the purpose of antiviral activity, the autophagic mechanism induced by Fdb could destroy the viral replicative site through autophagosome function, leading to the possibility of the effectiveness of IFN-α to suppress viral activity during the acute period of severe viral infection. The induced autophagy phenomenon focused upon in this study can be a strategy to lead cells to a suboptimal condition (reduced metabolism from overactive viral replication) for the so-called “pro-viral” mechanism that inhibits the virus life cycle and promotes cell survival conditions (“pro-cell”) to fight against the virus. Similarly, Fdb treatment with a suboptimal dose can reduce drug toxicity in both normal and cancer cells, but the cells showed a difference in their ability to induce the mediators of cell survival, such as Beclin-1 (anti-apoptosis), found downstream of mTOR and LC3B. Thus, the combination of Fdb with IFN-α for cancer treatment benefits from both the direct properties of IFN-α and the indirect inhibition of the tumor cells via the immune system.

In conclusion, an appropriate dose of Fdb can induce autophagy that induces pro-cell survival and may attenuate the pro-viral mechanism, leading to attenuation of IFN-α-induced STAT1 antiviral signals in virus-infected cells. The effectiveness of IFN-α and the detailed stimulation of the defensive activities of cells require further investigation. Studies of anticancer therapy require the evaluation of pro-cell survival that can prolong the life of normal cells in the body and have selective toxic effects on cancer cells during the period of chemotherapy by the different outcomes of autophagy response after treatment. This experimental model and investigative approach can be applied in order to magnify basic studies for the development of other antiviral and anticancer drugs.

This experiment was not aimed at evaluating the effectiveness of the drugs in order to reduce the replication of the virus in virally infected cells. The objective of this study was instead to define the mechanistic interaction of two drugs: Fdb, which stimulates the cells via adaptive autophagy, and IFN-α, which induces regulation of STAT1 signaling in certain cell types that are at a high risk of induced death during chemotherapy that kills the cancer cells or during antiviral drug treatment that kills infected cells as well as normal cells. Hence, the combined effect between the two factors within living cells is of benefit to the effective regulation of the body’s immune system during therapeutic manipulation against cancer and/or the viral infection.

## Data Availability

Not applicable.
